# HIV research output in African Countries between 1986–2020

**DOI:** 10.1371/journal.pgph.0000544

**Published:** 2023-06-22

**Authors:** Mukhtar A. Ijaiya, Adebanjo Olowu, Habibat A. Oguntade, Seun Anjorin, Olalekan A. Uthman

**Affiliations:** 1 Jhpiego, Abuja, FCT, Nigeria; 2 Department of Epidemiology and Community Health, University of Minnesota School of Public Health, Minneapolis, MN, United States of America; 3 Warwick Centre for Applied Health Research and Delivery (WCAHRD), Division of Health Sciences, Warwick Medical School, University of Warwick, Coventry, United Kingdom; PLOS: Public Library of Science, UNITED STATES

## Abstract

HIV literature has grown exponentially since it was named the virus that causes acquired immunodeficiency syndrome (AIDS). Bibliometric analysis is a practical approach for quantitatively and qualitatively assessing scientific research. This work aims to describe HIV research output in Africa by country from 1986 until 2020. We conducted a search of the PubMed database in June 2021 for a 35-year period spanning 1986 to 2020. We comparatively weighed for countries’ populations, gross domestic product (GDP), and the number of persons living with HIV (PLHIV) by calculating the ratio of the number of publications from each country. We used Poisson regression models to explore the trends in countries’ HIV research output over the study period. The Pearson correlation analysis assessed the association between research output, population size, GDP, and the number of PLHIV.A total of 83,527 articles from African countries on HIV indexed in PubMed were included for analysis. Republic of South Africa, Uganda, Kenya, and Nigeria account for 54% of the total indexed publications with 33.2% (26,907); 8.4% (7,045); 7.3% (6,118); and 5.1% (4,254), respectively. Africa’s proportion of the world’s total HIV publications increased from 5.1% in 1986 to 31.3% in 2020. There was a strong positive and statistically significant correlation between the total indexed HIV publications and countries’ GDP (r = 0.59, P<0.01), population (r = 0.58, P<0.01), and the estimated number of PLHIV (r = 0.72, P<0.01). The study found that Africa’s contribution to global HIV research output increased over the 35 years, but it remains relatively low compared to the continent’s burden of HIV infections. Our findings also revealed major differences in research output across sub-regions in Africa, with the Republic of South Africa having the highest output. The factors associated with HIV research output were economic strength, disease epidemiology, and population size.

## Introduction

The burden of the Human immune deficiency virus (HIV) and acquired immunodeficiency syndrome (AIDS) remains a major public health concern in Africa. The HIV epidemic disproportionately impacts Africa compared to other regions. Of the 37.7 million global estimates of people living with HIV (PLHIV) in 2020, over 25.3 million are in Africa [1, 2]. Over half of the global new HIV infections were reported in Africa in 2020 [1–3]. In addition, several economic (e.g., poverty, famine, unemployment, child, and adult sex work, low educational attainment), political (e.g., corruption, internal conflicts, and refugee status), social (e.g., lack of recreational facilities, childhood marriage, gender imbalances, polygamy), and behavioral factors (e.g., risk-taking, circumcision, female genital cutting, alcohol, and substance misuse, gender-based violence) that have contributed to the spread of HIV in Africa have been well documented [4–7].

HIV literature has grown exponentially since it was named the virus that causes acquired immune deficiency syndrome [8, 9]. This growth is reflective of the increase in the number of HIV infections reported to the World Health Organization [9]. Research has provided an overview of the history of HIV literature and its growth, from the lack of standard terminology during the early eighties to the current standardized Medical Subject Headings (MeSH) terminology [10]. Through previous studies, HIV topic areas, leading institutions, and scientists have been identified [10].

Bibliometric analyses remain a crucial source of information about research work quantity and quality [11–15]. It allows investigators to assess trends in specific research areas by highlighting key characteristics of research products [16]. Essentially, bibliometric analysis helps to answer questions such as; who is doing the research? Where is the research being conducted? [16]. Bibliometric studies have also been used to identify leading journals, countries, and subject fields [10].

The advancement of science through the exchange and recognition of research is the primary argument supporting the use of bibliometrics [9]. Bibliometric analysis has also proven helpful in assisting decision-makers in identifying research gaps and interests for public policy planning, funding, and implementation [17]. Bibliometric analysis results, however, do not provide the contextual impact of included studies and have limited usefulness for cross-specialization/topic area comparisons. [18].

Bibliometric studies of HIV research have described the distribution and variation in scientific output over time [9]. For example, an analysis of the number of medical publications relative to indicators in 2003 showed that countries with the highest number of PLHIV are not leading the research output on HIV [19]. Furthermore, it has been found that only the USA and Western Europe accounted for 83% of the global HIV research output between 1986 and 2003 in three journals [8]. In addition, the five most developed regions of the nine world regions accounted for almost all (92%) of the world’s research output on HIV [8].

The year 1986 represents an important point in the history of HIV. In 1986, The International Committee on the Taxonomy of Viruses announced HIV as the official term for the virus that causes AIDS [20, 21]. This work aims to describe HIV research output in Africa by country, beginning when HIV was first announced as the official term for the virus that causes AIDS. In addition, this analysis will also serve as an update of previous bibliometric analyses of HIV research in Africa [8, 9, 22].

## Methods

### Data sources

We searched the PubMed database in June 2021 in order to obtain the HIV research output by volume of each African country over a 35 years period (January 1, 1986, to December 31, 2020). The search was conducted by specifying the search parameters: search terms, place, and year of publication. The search terms used are detailed in Table 1. The country names were imputed in their different possible spelling formats, for example, "Cape Verde" = "Cape Verde OR Cabo Verde". We also considered that some country names double as names of parts of other countries; for example, Benin and Niger are names of African Countries and places in Nigeria. To address potential errors that may arise from this nomenclature, appropriate search command restrictions were written: "Benin" = "Benin NOT Nigeria" and "Niger" = "Niger NOT Nigeria."

**Table 1:** Key search terms for PubMed database.

	Search Terms
**PubMed**	HIV Infections [MeSH] OR HIV Infections* OR HIV [MeSH] OR HIV* OR hiv-1 [MeSH] OR hiv-1* OR hiv-2 [MeSH] OR hiv-2* OR hiv1 [MeSH] OR hiv1* OR hiv2 [MeSH] OR hiv2* OR human immunodeficiency virus [MeSH] OR human immunodeficiency virus* OR human immunedeficiency virus [MeSH] OR human immunedeficiency virus* OR human immuno-deficiency virus [MeSH] OR human immuno-deficiency virus* OR human immune-deficiency virus [MeSH] OR human immune-deficiency virus* OR acquired immunodeficiency syndrome [MeSH] OR acquired immunodeficiency syndrome* OR acquired immunedeficiency syndrome [MeSH] OR acquired immunedeficiency syndrome* OR acquired immuno-deficiency syndrome [MeSH] OR acquired immuno-deficiency syndrome* OR acquired immune-deficiency syndrome [MeSH] OR acquired immune-deficiency syndrome* OR AIDS [MeSH] OR AIDS*

*Any variation in the word will be included in the search

The volume of HIV research articles indexed in the PubMed database over the study period was used as a proxy for HIV total research output in Africa. The total number of publications was the total number of all published articles returned from the search indexed on PubMed. A comparative weighting for population, Gross Domestic Product (GDP), and number of Persons Living with HIV (PLHIV) was done by calculating the ratio of articles from each country. These variables were selected for inclusion in the analysis based on the availability of reliable data throughout the study period. The population and GDP estimates were obtained from the World Development Indicators database of The World Bank, while the PLHIV burden estimates among adults and children (all ages) were obtained from the UNAIDS Global database on HIV epidemiology and response [2, 23]. As a result, four top 10 lists of countries with the highest absolute number of publications relative to population, GDP, and number of PLHIV, respectively, were created. HIV research output was also analyzed at the geographical sub-regional level. Countries were grouped into five geographical sub-regions: Eastern, Middle, Northern, Southern, and Western Africa, following the United Nations geo-scheme.

### Statistical analyses

We used Poisson regression models to explore the publication output time trends over the 35 years period, with the absolute number of publications as the outcome variable and the year of publication as the predictor variable. The Poisson regression fits the model below:

ln(p)=a+β*(t)

where

p equals the number of articles per year,

log is the natural log; a is the intercept,

β is the trend, and

t is the year–year starting from 1986.

Average annual percentage changes (AAPC) were calculated at country and sub-regional levels using the formula: AAPC = 100 * (exp (β) - 1).

The absolute and relative increases in the number of publications between the beginning and end of the search period were also calculated at both country and sub-regional levels:

$$‐Percent\;relative\;growth=(\frac{number\;of\;publications\;in\;2020}{\left(number\;of\;publications\;in\;2020-number\;of\;publications\;in\;1986\right)})\;X\;100$$

Further analysis of the change in trend per year was conducted at country and sub-regional levels by assessing change over the seven 5-year periods. In addition, the proportion of Africa’s research output in HIV of the entire world HIV research output was calculated for each year as below:

$$‐Annual\;Percent\;share\;of\;world\;HIV\;research\;output=(\frac{number\;of\;HIV\;articles\;on\;Africa}{total\;number\;of\;HIV\;articles\;indexed\;in\;PubMed})\;X\;100$$

Using Pearson correlation analysis, we explored the association between research output and population size, research output and GDP, research output, and the number of PLHIV. We log-transformed the number of publications, population size, GDP, and number of PLHIV to linearize these associations. We used the PubMedR package to search and collate the metadata of publications and articles gathered from the PubMed database. Data were processed and analyzed with R, a language and environment for statistical computing [24].

## Results

A total of 83,527 articles on HIV indexed in PubMed between 1986 and 2020 were included for analysis. Countries in East Africa accounted for 39% of the total indexed publications, followed closely by countries in Southern Africa, with 36% of the total indexed publications. Countries in these two regions and countries in West Africa (16%) collectively account for 90% of all indexed publications on HIV. Conversely, the Republic of South Africa in the Southern Africa sub-region had the highest research productivity by volume, accounting for 32% of all publications in Africa, with the proportion of indexed articles from the following five countries across East (Uganda-8%; Kenya-7%; Tanzania- 5%, Malawi-4%) and West Africa (Nigeria-5%) accounting for less than Republic of South Africa’s share at 30%. Sao Tome and Principe (8) had the least indexed articles.

Within each sub-region, Uganda (22%), Cameroon (37%), Western Sahara (31%), Republic of South Africa (89%), and Nigeria (33%) represent countries with the highest number of indexed articles in Eastern, Middle, Northern, Southern and Western Africa respectively (Table 2). When analyzed by each country’s income category, 8 Upper middle-income countries (UMICs) account for 36% of the total indexed publications. In comparison, 23 lower-middle-income countries (LMICs) and 25 low-income countries (LICs) had 33% and 31% of total indexed publications, respectively. The only high-income country (HIC) on the list (Seychelles Island) had just 20 indexed publications over the study period.

**Table 2:** Number of articles indexed by PubMed from African countries, income group, percentage of sub-regional and African publications.

Sub-Region	Country	Income Group	Quintile	Total Indexed Publications	Proportion of Indexed Publications in Sub-region	Proportion of Indexed Publications in Africa
**Eastern Africa**	Burundi	LIC	69 to 318	214	0.7%	0.3%
	Comoros	LMIC	8 to 68	25	0.1%	0.0%
	Djibouti	LMIC	8 to 68	63	0.2%	0.1%
	Eritrea	LIC	8 to 68	47	0.1%	0.1%
	Ethiopia	LIC	1496 to 26907	2,965	9.2%	3.5%
	Kenya	LMIC	1496 to 26907	6,118	19.0%	7.3%
	Madagascar	LIC	69 to 318	148	0.5%	0.2%
	Malawi	LIC	1496 to 26907	3,483	10.8%	4.2%
	Mauritius	UMIC	8 to 68	58	0.2%	0.1%
	Mayotte	LIC	8 to 68	27	0.1%	0.0%
	Mozambique	LIC	686 to 1495	1,104	3.4%	1.3%
	Reunion	LIC	8 to 68	44	0.1%	0.1%
	Rwanda	LIC	686 to 1495	1,155	3.6%	1.4%
	Seychelles Island	HIC	8 to 68	20	0.1%	0.0%
	Somalia	LIC	69 to 318	93	0.3%	0.1%
	Uganda	LIC	1496 to 26907	7,045	21.9%	8.4%
	United Republic of Tanzania	LIC	1496 to 26907	3,813	11.9%	4.6%
	Zambia	LMIC	1496 to 26907	2,768	8.6%	3.3%
	Zimbabwe	LMIC	1496 to 26907	2,980	9.3%	3.6%
**Eastern Africa Total**				**32,170**		**38.5%**
**Middle Africa**	Angola	LMIC	69 to 318	137	2.6%	0.2%
	Cameroon	LMIC	1496 to 26907	1,940	37.1%	2.3%
	Central African Republic	LIC	319 to 685	341	6.5%	0.4%
	Chad	LIC	69 to 318	88	1.7%	0.1%
	Congo	LMIC	686 to 1495	1,323	25.3%	1.6%
	Democratic Republic of the Congo	LIC	686 to 1495	937	17.9%	1.1%
	Equatorial Guinea	UMIC	8 to 68	57	1.1%	0.1%
	Gabon	UMIC	319 to 685	398	7.6%	0.5%
	Sao Tome and Principe	LMIC	8 to 68	8	0.2%	0.0%
**Middle Africa Total**				**5,229**		**6.3%**
**Northern Africa**	Algeria	UMIC	8 to 68	63	2.3%	0.1%
	Egypt	LMIC	686 to 1495	813	29.2%	1.0%
	Libyan Arab Jamahiriya	UMIC	69 to 318	133	4.8%	0.2%
	Morocco	LMIC	69 to 318	311	11.2%	0.4%
	Sudan	LMIC	319 to 685	372	13.3%	0.4%
	Tunisia	LMIC	69 to 318	218	7.8%	0.3%
	Western Sahara	LMIC	686 to 1495	877	31.5%	1.0%
**Northern Africa Total**				**2,787**		**3.3%**
**Southern Africa**	Botswana	UMIC	1496 to 26907	1,591	5.3%	1.9%
	Lesotho	LMIC	319 to 685	406	1.3%	0.5%
	Namibia	UMIC	319 to 685	461	1.5%	0.6%
	Republic of South Africa	UMIC	1496 to 26907	26,907	88.8%	32.2%
	Swaziland	LMIC	686 to 1495	927	3.1%	1.1%
**Southern Africa Total**				**30,292**		**36.3%**
**Western Africa**	Benin	LIC	319 to 685	334	2.6%	0.4%
	Burkina Faso	LIC	686 to 1495	824	6.3%	1.0%
	Cape Verde	LMIC	319 to 685	340	2.6%	0.4%
	Cote d’Ivoire	LMIC	686 to 1495	1,169	9.0%	1.4%
	Gambia	LIC	319 to 685	438	3.4%	0.5%
	Ghana	LMIC	1496 to 26907	1,506	11.5%	1.8%
	Guinea	LIC	686 to 1495	1,451	11.1%	1.7%
	Guinea-Bissau	LIC	319 to 685	348	2.7%	0.4%
	Liberia	LIC	69 to 318	129	1.0%	0.2%
	Mali	LIC	319 to 685	493	3.8%	0.6%
	Mauritania	LMIC	8 to 68	40	0.3%	0.0%
	Niger	LIC	69 to 318	214	1.6%	0.3%
	Nigeria	LMIC	1496 to 26907	4,254	32.6%	5.1%
	Saint Helena	LMIC	8 to 68	34	0.3%	0.0%
	Senegal	LMIC	686 to 1495	975	7.5%	1.2%
	Sierra Leone	LIC	69 to 318	172	1.3%	0.2%
	Togo	LIC	319 to 685	328	2.5%	0.4%
**Western Africa Total**				**13,049**		**15.6%**
**Grand Total**				**83,527**		

*Mayotte and Reunion Islands are French overseas territories in Africa

HIC: High Income Country

LIC: Low-Income Country

LMIC: Lower Middle-Income Country

UMIC: Upper Middle-Income Country

**Fig 1:**
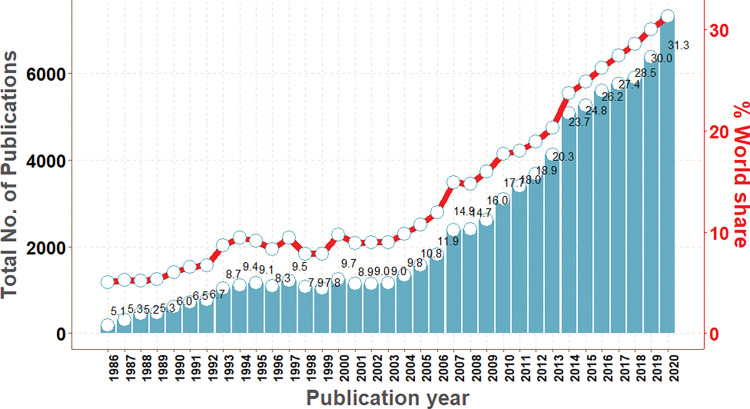
Africa’s annual percent share of global HIV research output, 1986–2020.

Not surprisingly, the last year (2020) of the study period had the most research output, with 7,310 indexed articles, as opposed to the first year (1986), with the least research output at 188 indexed articles. It was striking to note that Africa’s proportion of the world’s total HIV publications rose from 5.1% in 1986 to a record high of 31.3% in 2020, with Africa’s lowest proportions in 1986 (5.1%), 1988 (5.2%), 1987 (5.3%) and 1989 (5.3%) (Fig 1). Moreover, since 2005, Africa’s share of the world’s research output has seen a consistent and appreciable upward trend and has not gone below 10.8%.

**Fig 2:**
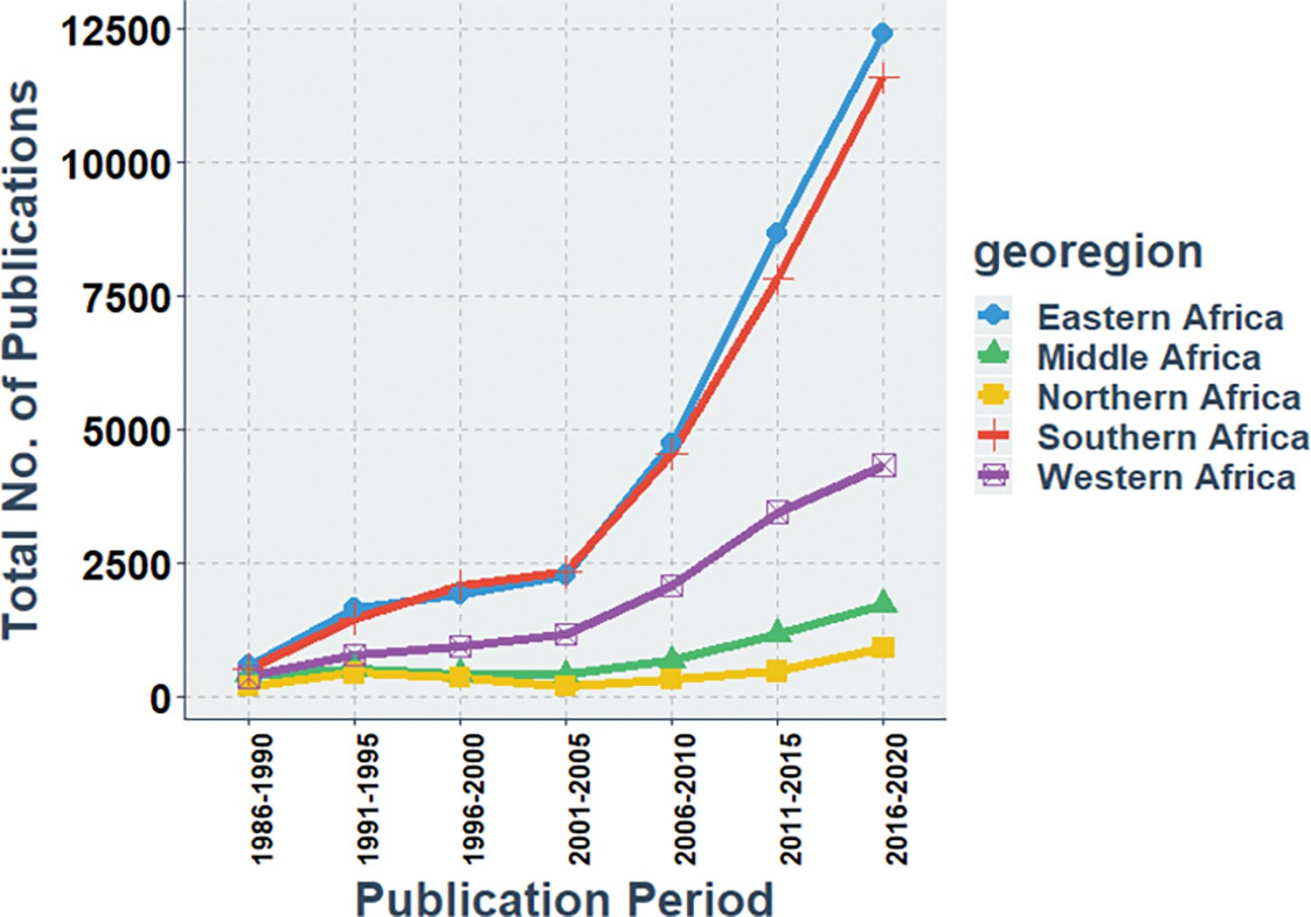
Sub-regional HIV research output trend in Africa, 1986–2020.

A similar trend is noted when research productivity trends are assessed on a sub-regional basis (Fig 2). The highest research productivity alternated between Eastern and Southern Africa before 2005; however, since 2005, there has been a marked upwards trend in research productivity in both sub-regions, with Eastern Africa (25,790 total indexed articles) slightly outperforming Southern Africa (23,939 total indexed articles) in the last fifteen years of the study period. The other three sub-regions have also seen an upwards trend in HIV research productivity to a lesser degree in the descending order: Western Africa, Middle Africa, and Northern Africa.

**Fig 3:**
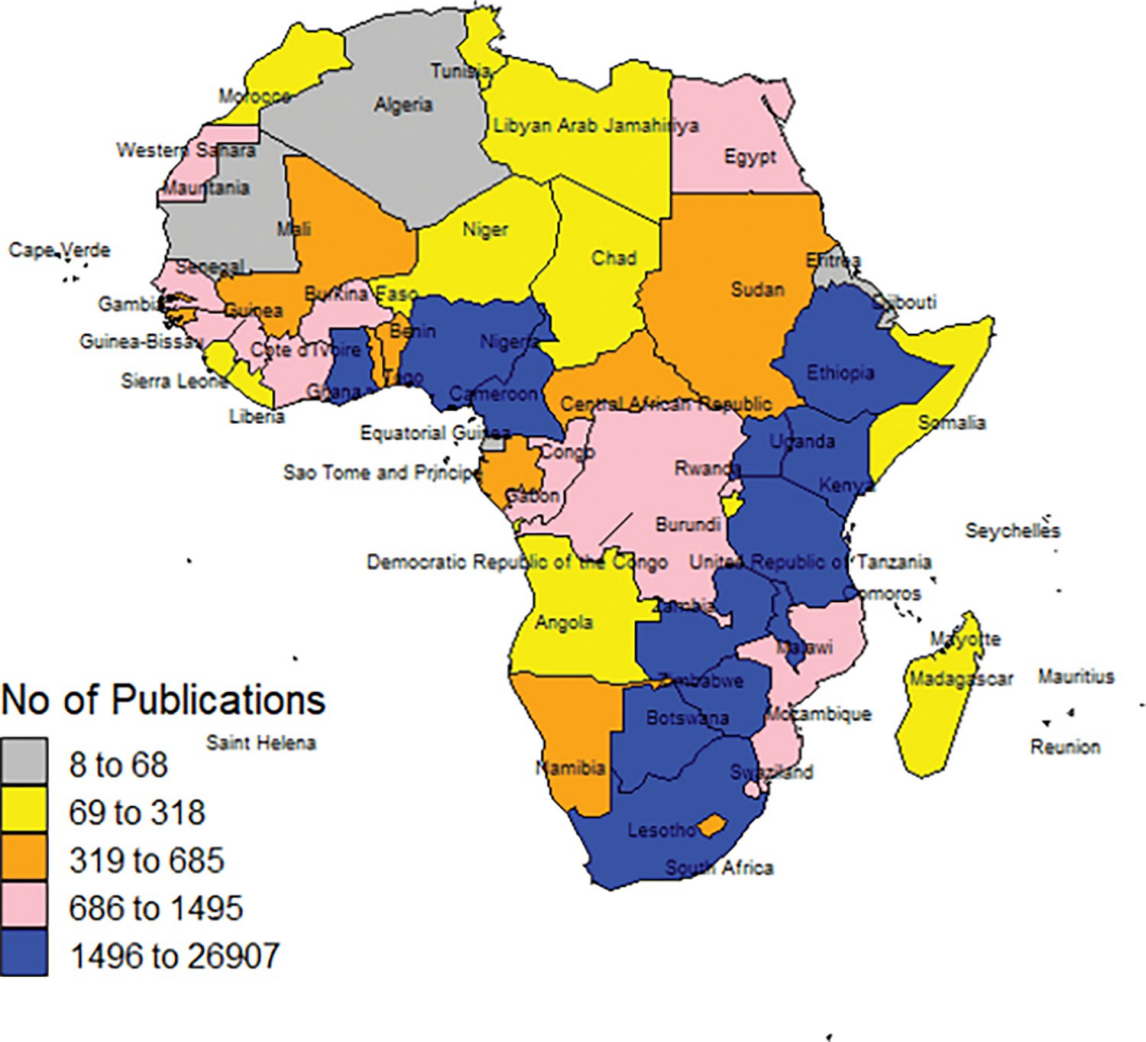
Color-coded Africa map representing the total number of publications per country by quintiles (https://github.com/nasa/World-Wind-Java/tree/master/WorldWind/testData/shapefiles; https://github.com/nasa/World-Wind-Java/blob/7c9886ab67ac03d53bdb04f161b9605d3f3dd810/WorldWind/NASA_Open_Source_Agreement_1.3.txt).

The Fig 3 map breaks down the total number of publications into statistically representative quintiles. Twelve countries that had between 1,496 to 26,907 indexed articles overall were in the highest quintile, followed by 11 countries with 686 to 1,495 indexed articles in the second quintile, then 11 countries with 319 to 685 indexed articles in the third quintile, 11 countries with 69 to 318 indexed articles in the fourth quintile and 12 countries with 8 to 68 indexed articles in the lowest quintile.

The ten top-ranking countries, which collectively represent 75% of all indexed publications for the study period, are shown in Table 3 below. All the countries had more indexed articles in the last year of the study period than in the first year, with positive absolute and relative growth. Notably, Ethiopia, Malawi, and Zimbabwe had no indexed publications at the beginning of the study period. In addition, each country’s relative share of indexed publications on HIV increased between the beginning of the study period (1986) and the end of the study period (2020). There was also a continuous increase in research output, with a statistically significant average annual percentage change among these ten countries, ranging from 8.4 for Zambia to 14.8 for Ethiopia.

**Table 3:** Top 10 Countries by HIV articles, proportion of Africa’s publications, relative growth, and annual percent change: 1986–2020.

Country	Total Indexed Publications 1986–2020	Total Indexed Publications 1986	Total Indexed Publications 2020	Absolute growth (%)	Relative growth (%)	AAPC	p-value
**Cameroon**	1,940 (2.3)	2 (1.1)	159 (2.2)	7950	7850	10.4 (9.8 to 11.0)	<0.001
**Zambia**	2,768 (3.3)	5 (2.7)	211 (2.9)	4220	4120	8.4 (7.9 to 8.9)	<0.001
**Ethiopia**	2,965 (3.5)	0 (0.0)	489 (6.7)	Inf	Inf	14.8 (14.2 to 15.4)	<0.001
**Zimbabwe**	2,980 (3.6)	0 (0.0)	262 (3.6)	Inf	Inf	8.6 (8.1 to 9.0)	<0.001
**Malawi**	3,483 (4.2)	0 (0.0)	320 (4.4)	Inf	Inf	11.5 (11.0 to 12.0)	<0.001
**United Republic of Tanzania**	3,813 (4.6)	6 (3.2)	297 (4.1)	4950	4850	8.8 (8.4 to 9.2)	<0.001
**Nigeria**	4,254 (5.1)	3 (1.6)	417 (5.7)	13900	13800	11.3 (10.9 to 11.8)	<0.001
**Kenya**	6,118 (7.3)	8 (4.3)	556 (7.6)	6950	6850	11.0 (10.6 to 11.4)	<0.001
**Uganda**	7,045 (8.4)	10 (5.3)	595 (8.1)	5950	5850	10.2 (9.8 to 10.5)	<0.001
**Republic of South Africa**	26,907 (33.2)	32 (17.0)	2,330 (31.9)	7281.3	7181.3	9.5 (9.4 to 9.7)	<0.001

AAPC: Average Annual Percentage Change

Inf: Infinity

At the sub-regional level, every region also had a higher number of indexed articles at the end of the study than at the beginning, with a positive absolute and relative growth (Table 4). However, their relative share of indexed publications between the beginning and end of the study period varied. For example, the relative share of the Eastern and Southern African regions increased, while that of Middle, Northern, and Western(marginally) Africa decreased. Similarly, there was a continuous increase in research output with a statistically significant average annual percentage change among these sub-regions, ranging from 4.2 for Northern Africa to 10.2 for Eastern Africa.

**Table 4:** Sub-regional total number of HIV articles, proportion of Africa’s publications, relative growth, and annual percent change: 1986–2020.

Sub-region	Total Indexed Publications 1986–2020	Total Indexed Publications 1986	Total Indexed Publications 2020	Absolute growth (%)	Relative growth (%)	AAPC	p-value
**Eastern Africa**	32,170 (39.0)	42 (22.0)	2,978 (41.0)	7090.5	6990.5	10.1 (9.9 to 10.2)	<0.001
**Middle Africa**	5,229 (6.0)	72 (38.0)	380 (5.0)	527.8	427.8	5.8 (5.5 to 6.1)	<0.001
**Northern Africa**	2,787 (3.0)	9 (5.0)	205 (3.0)	2277.8	2177.8	4.2 (3.8 to 4.6)	<0.001
**Southern Africa**	30,292 (36.0)	36 (19%)	2,712 (37.0)	7533.3	7433.3	9.8 (9.7 to 10.0)	<0.001
**Western Africa**	13,049 (16.0)	29 (15.0)	1,035 (14.0)	3569.0	3469.0	8.3 (8.1 to 8.5)	<0.001

AAPC: Average Annual Percentage Change

**Fig 4:**
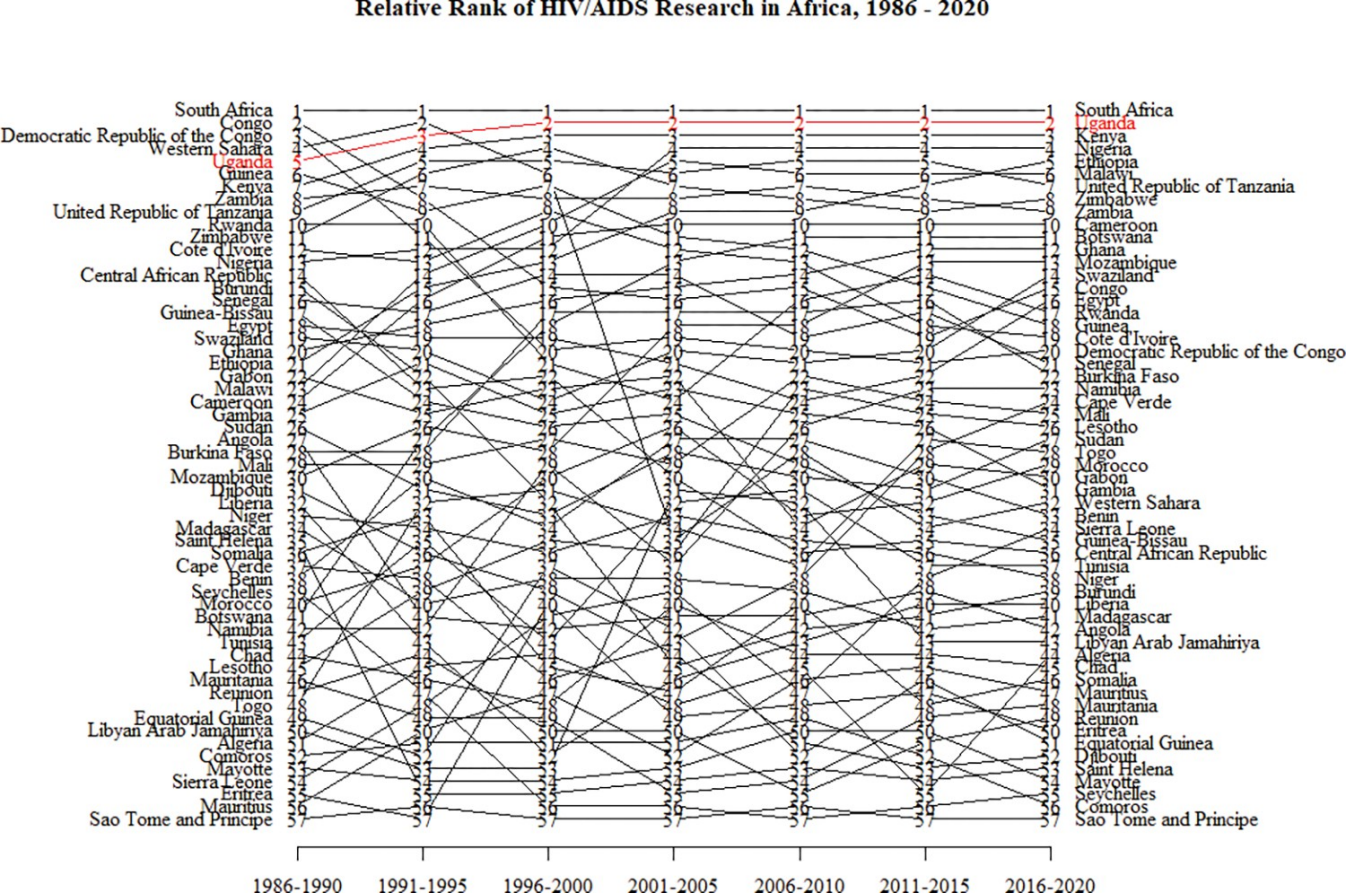
Relative rank of countries in HIV research productivity in Africa, 1986–2020.

Results from an analysis of each country’s HIV research productivity across seven different five-year periods are detailed in Fig 4. The Republic of South Africa ranked 1^st^ across all the time bands. Congo, the Democratic Republic of Congo, and Western Sahara ranked 2^nd^, 3^rd,^ and 4^th^, respectively, in the first-time band (1986–1990), markedly dropping to 15^th^, 20^th^, and 32^nd^ positions in the last-time band (2016–2020). Uganda’s relative rank improved from 5^th^ in the 1986–1990 time band to 2^nd^ in the 2016–2020 time band. Kenya, Nigeria, and Ethiopia, whose relative ranks at the 2016–2020 time band were 3^rd^, 4^th^, and 5^th^, were hitherto at the 7^th^, 13^th^, and 21^st^ positions, respectively, at the time band corresponding to the beginning of the study period.

The five bottom-ranked countries at the time band corresponding to the beginning of the study period did not see any appreciable improvement in relative rank across the time bands, except for Sierra Leone, whose relative rank improved from 54^th^ to 34^th^. The other four countries, Mayotte Island (53^rd^), Eritrea (55^th^), Mauritius (56^th^), and Sao Tome and Principe Island (57^th^), either changed marginally or maintained their relative positions- at 54^th^, 50^th^, 47^th^, and 57^th^, respectively in the end of study period time band. In addition, while both Mayotte and Sao Tome and Principe Islands remained among the bottom five countries at the end of the study period time band, Saint Helena (53^rd^), Seychelles (55^th^), and Comoros Islands (56^th^) were also in the bottom five during this time band, and dropped from their relative ranks of 35^th^, 39^th^ and 52^nd^ at the beginning of study time band.

**Fig 5:**
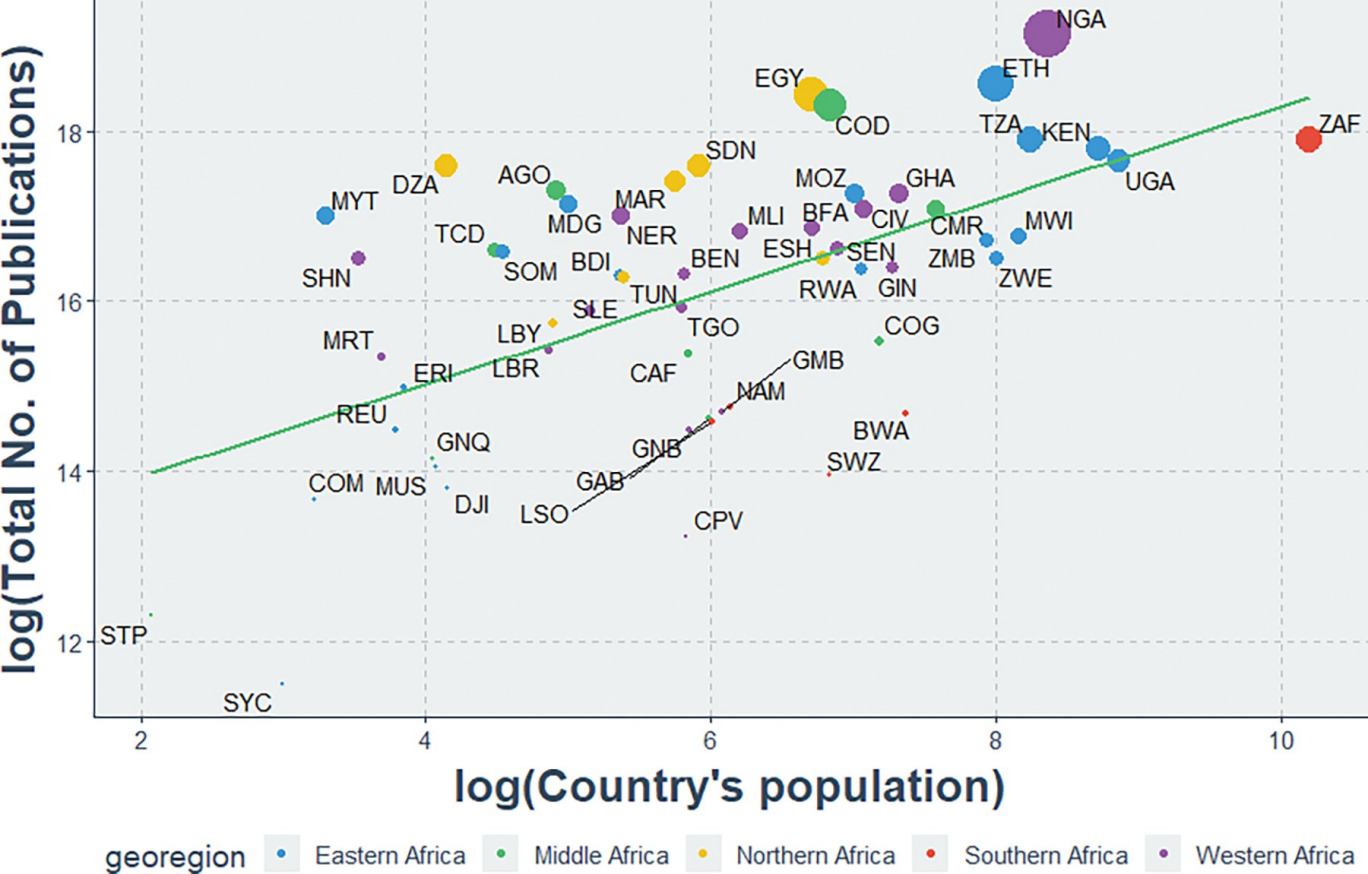
Association between total HIV publications and the population of each African country. AGO: Angola; BDI: Burundi; BEN: Benin; BFA: Burkina Faso; BWA: Botswana; CAF: Central African Republic; CIV: Cote d’Ivoire; CMR: Cameroon; COD: Democratic Republic of the Congo; COG: Congo; COM: Comoros; CPV: Cape Verde; DJI: Djibouti; DZA: Algeria; EGY: Egypt; ERI: Eritrea; ESH: Western Sahara; ETH: Ethiopia; GAB: Gabon; GHA: Ghana; GIN: Guinea; GMB: Gambia; GNB: Guinea-Bissau; GNQ: Equatorial Guinea; KEN: Kenya; LBR: Liberia; LBY: Libyan Arab Jamahiriya; LSO: Lesotho; MAR: Morocco; MDG: Madagascar; MLI: Mali; MOZ: Mozambique; MRT: Mauritania; MUS: Mauritius; MWI: Malawi; MYT: Mayotte; NAM: Namibia; NER: Niger; NGA: Nigeria; REU: Reunion; RWA: Rwanda; SDN: Sudan; SEN: Senegal; SHN: Saint Helena; SLE: Sierra Leone; SOM: Somalia; STP: Sao Tome and Principe; SWZ: Swaziland; SYC: Seychelles; TCD: Chad; TGO: Togo; TUN: Tunisia; TZA: United Republic of Tanzania; UGA: Uganda; ZAF: South Africa; ZMB: Zambia; ZWE: Zimbabwe.

A strong positive and statistically significant correlation existed between the total indexed HIV publications and countries’ populations (r = 0.58, P<0.01). Populous countries like Ethiopia, Kenya, Nigeria, the Republic of South Africa, Tanzania, and Uganda had the most research output compared to their respective populations. Conversely, much less populous countries like Comoros, Sao Tome & Principe, and Seychelles Islands had the least research output compared to their respective populations (Fig 5).

**Fig 6:**
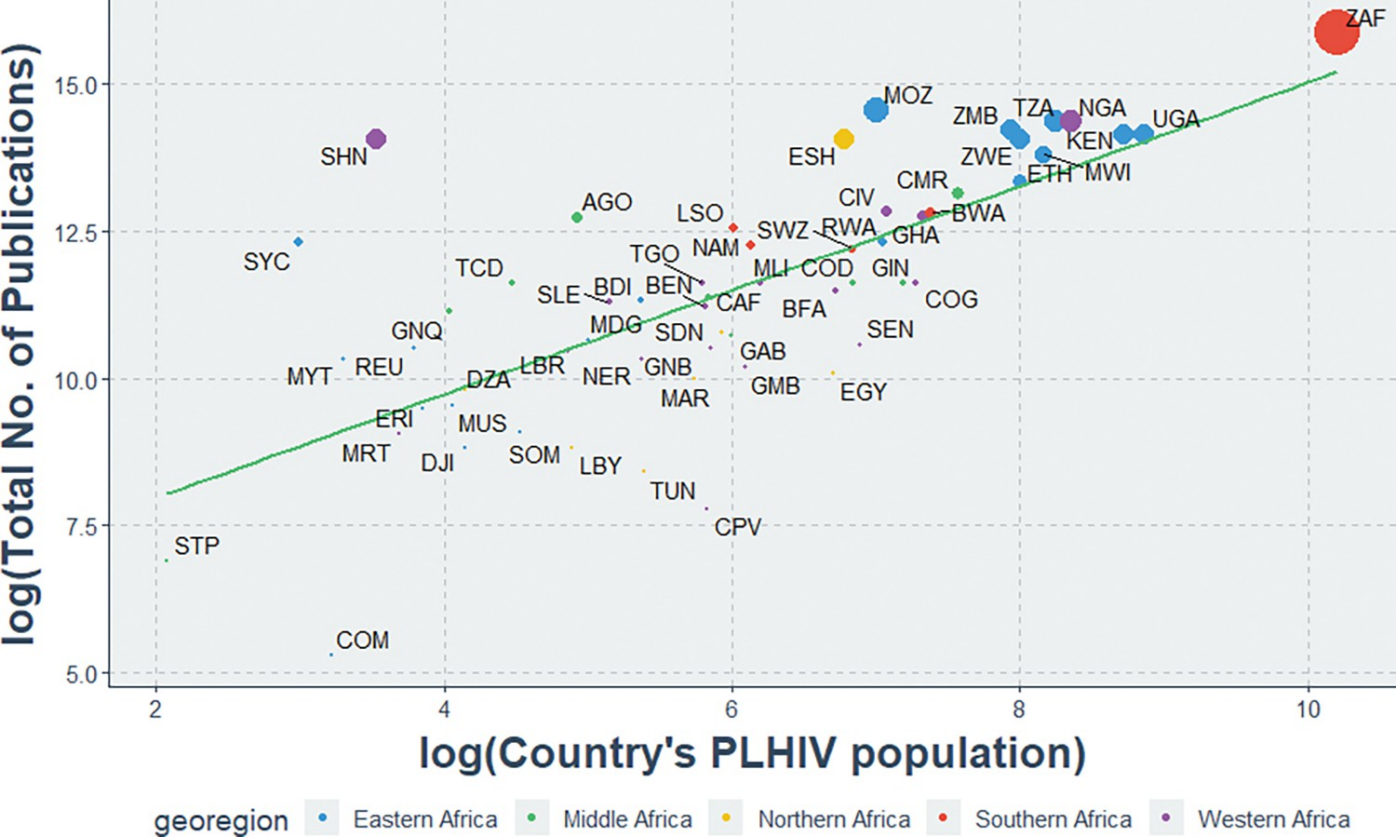
Association between total HIV publications and the PLHIV population of each African country. AGO: Angola; BDI: Burundi; BEN: Benin; BFA: Burkina Faso; BWA: Botswana; CAF: Central African Republic; CIV: Cote d’Ivoire; CMR: Cameroon; COD: Democratic Republic of the Congo; COG: Congo; COM: Comoros; CPV: Cape Verde; DJI: Djibouti; DZA: Algeria; EGY: Egypt; ERI: Eritrea; ESH: Western Sahara; ETH: Ethiopia; GAB: Gabon; GHA: Ghana; GIN: Guinea; GMB: Gambia; GNB: Guinea-Bissau; GNQ: Equatorial Guinea; KEN: Kenya; LBR: Liberia; LBY: Libyan Arab Jamahiriya; LSO: Lesotho; MAR: Morocco; MDG: Madagascar; MLI: Mali; MOZ: Mozambique; MRT: Mauritania; MUS: Mauritius; MWI: Malawi; MYT: Mayotte; NAM: Namibia; NER: Niger; NGA: Nigeria; REU: Reunion; RWA: Rwanda; SDN: Sudan; SEN: Senegal; SHN: Saint Helena; SLE: Sierra Leone; SOM: Somalia; STP: Sao Tome and Principe; SWZ: Swaziland; SYC: Seychelles; TCD: Chad; TGO: Togo; TUN: Tunisia; TZA: United Republic of Tanzania; UGA: Uganda; ZAF: South Africa; ZMB: Zambia; ZWE: Zimbabwe.

Similarly, there was a stronger positive correlation between the total indexed HIV publications and the number of PLHIV (r = 0.72, P<0.01). From Fig 6 below, countries with the largest PLHIV populations (Ethiopia, Kenya, Nigeria, Republic of South Africa, Tanzania, and Uganda) had higher research output compared to countries with the smallest PLIHIV numbers (Comoros, Sao Tome & Principe, and Seychelles Islands).

**Fig 7:**
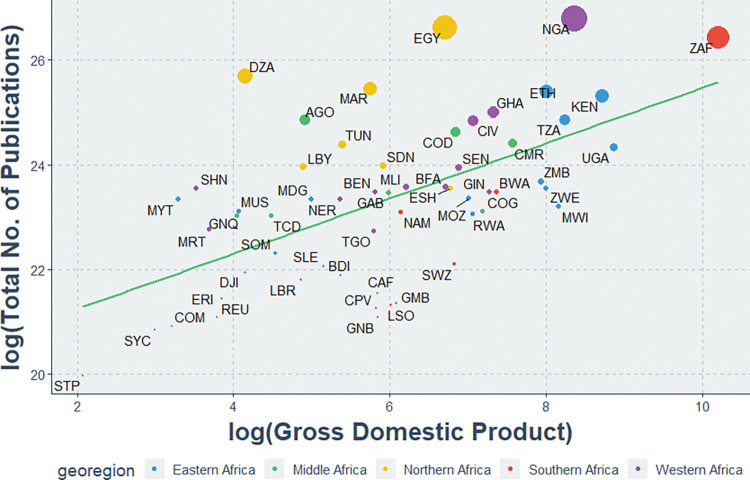
Association between total HIV publications and the GDP of each African country. AGO: Angola; BDI: Burundi; BEN: Benin; BFA: Burkina Faso; BWA: Botswana; CAF: Central African Republic; CIV: Cote d’Ivoire; CMR: Cameroon; COD: Democratic Republic of the Congo; COG: Congo; COM: Comoros; CPV: Cape Verde; DJI: Djibouti; DZA: Algeria; EGY: Egypt; ERI: Eritrea; ESH: Western Sahara; ETH: Ethiopia; GAB: Gabon; GHA: Ghana; GIN: Guinea; GMB: Gambia; GNB: Guinea-Bissau; GNQ: Equatorial Guinea; KEN: Kenya; LBR: Liberia; LBY: Libyan Arab Jamahiriya; LSO: Lesotho; MAR: Morocco; MDG: Madagascar; MLI: Mali; MOZ: Mozambique; MRT: Mauritania; MUS: Mauritius; MWI: Malawi; MYT: Mayotte; NAM: Namibia; NER: Niger; NGA: Nigeria; REU: Reunion; RWA: Rwanda; SDN: Sudan; SEN: Senegal; SHN: Saint Helena; SLE: Sierra Leone; SOM: Somalia; STP: Sao Tome and Principe; SWZ: Swaziland; SYC: Seychelles; TCD: Chad; TGO: Togo; TUN: Tunisia; TZA: United Republic of Tanzania; UGA: Uganda; ZAF: South Africa; ZMB: Zambia; ZWE: Zimbabwe.

There was a positive correlation between the total indexed HIV publications and countries’ GDP (r = 0.59, P<0.01) (Fig 7). Same countries: Ethiopia, Kenya, Nigeria, Republic of South Africa, Tanzania, and Uganda, with large populations, had the most research output when compared with GDP, and much less populous countries like Comoros, Sao Tome & Principe, and Seychelles Islands had the least research output when compared with GDP. A positive correlation was also between countries’ GDP and population size (r = 0.84, P<0.01).

**Fig 8:**
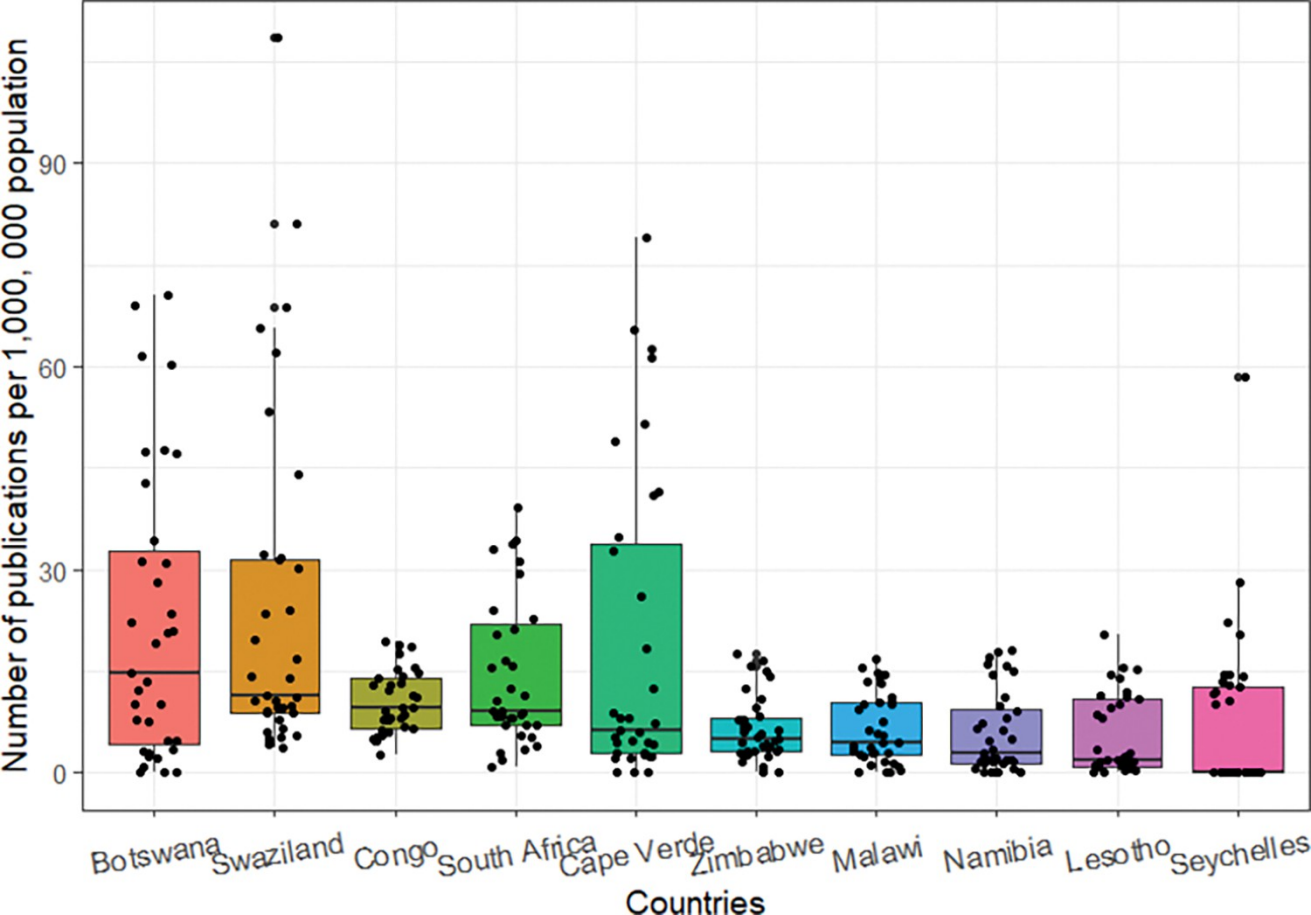
Top 10 most productive countries and the annual number of publications per 1,000,000 population, 1986–2020.

The 10-top ranked most productive countries when total HIV-indexed publications are considered per million inhabitants are in Fig 8 below. Malawi, the Republic of South Africa, and Zimbabwe, among the top 10 countries with the highest indexed publications, are also very productive per million inhabitants. In addition, much less populous countries like Cape Verde, Lesotho, and Seychelles Islands also had higher research output per million inhabitants.

**Fig 9:**
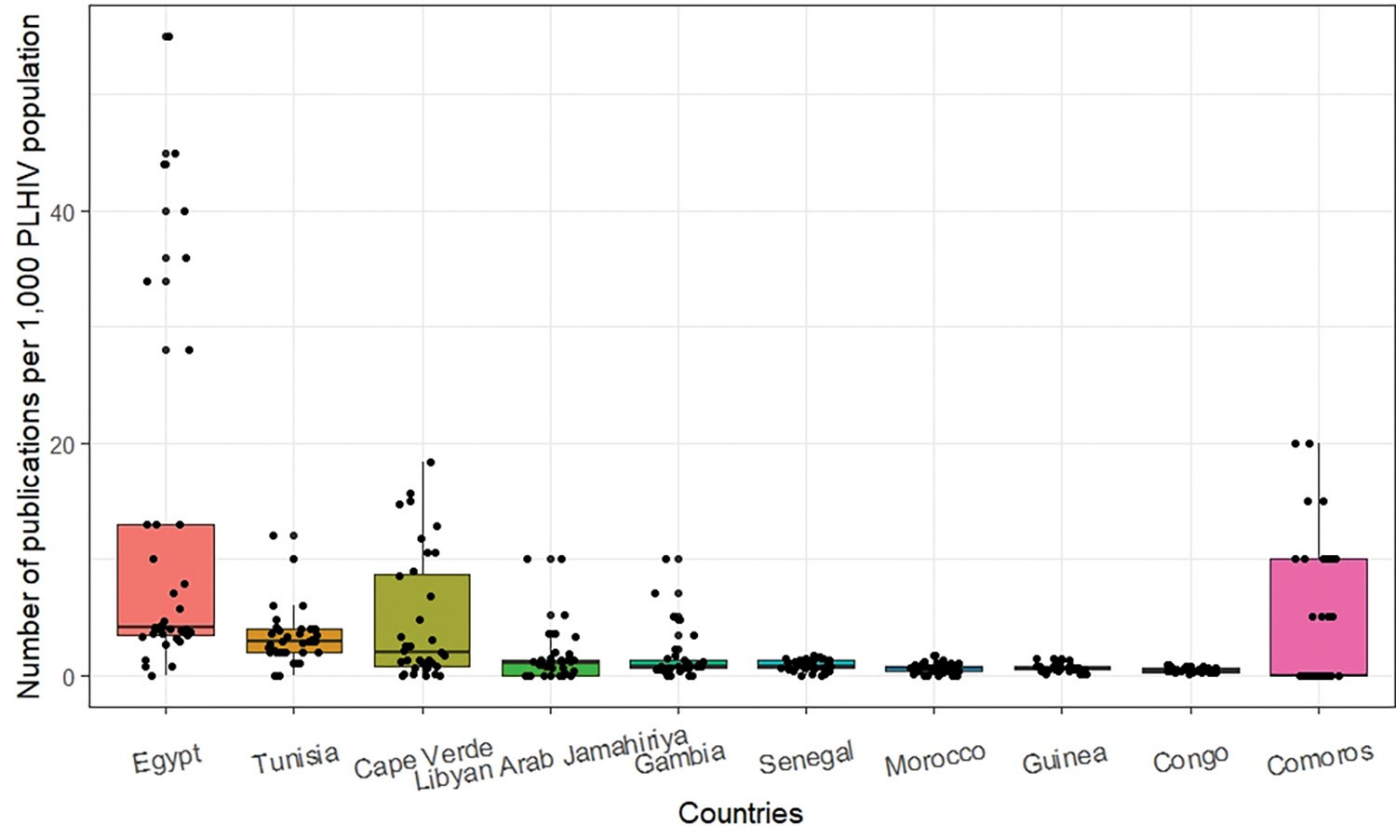
Top 10 most productive countries and the annual number of publications per 1,000 PLHIV population, 1986–2020.

The top 10 most productive countries when total HIV-indexed publications are compared per 1,000 PLHIV are illustrated in Fig 9. Countries with much smaller PLHIV populations were found to be the most productive. There were four Northern African countries: Egypt, Libyan Arab Jamahiriya, Morocco, and Tunisia. The remaining on the top 10 list were much smaller countries apart from Guinea and Senegal.

**Fig 10:**
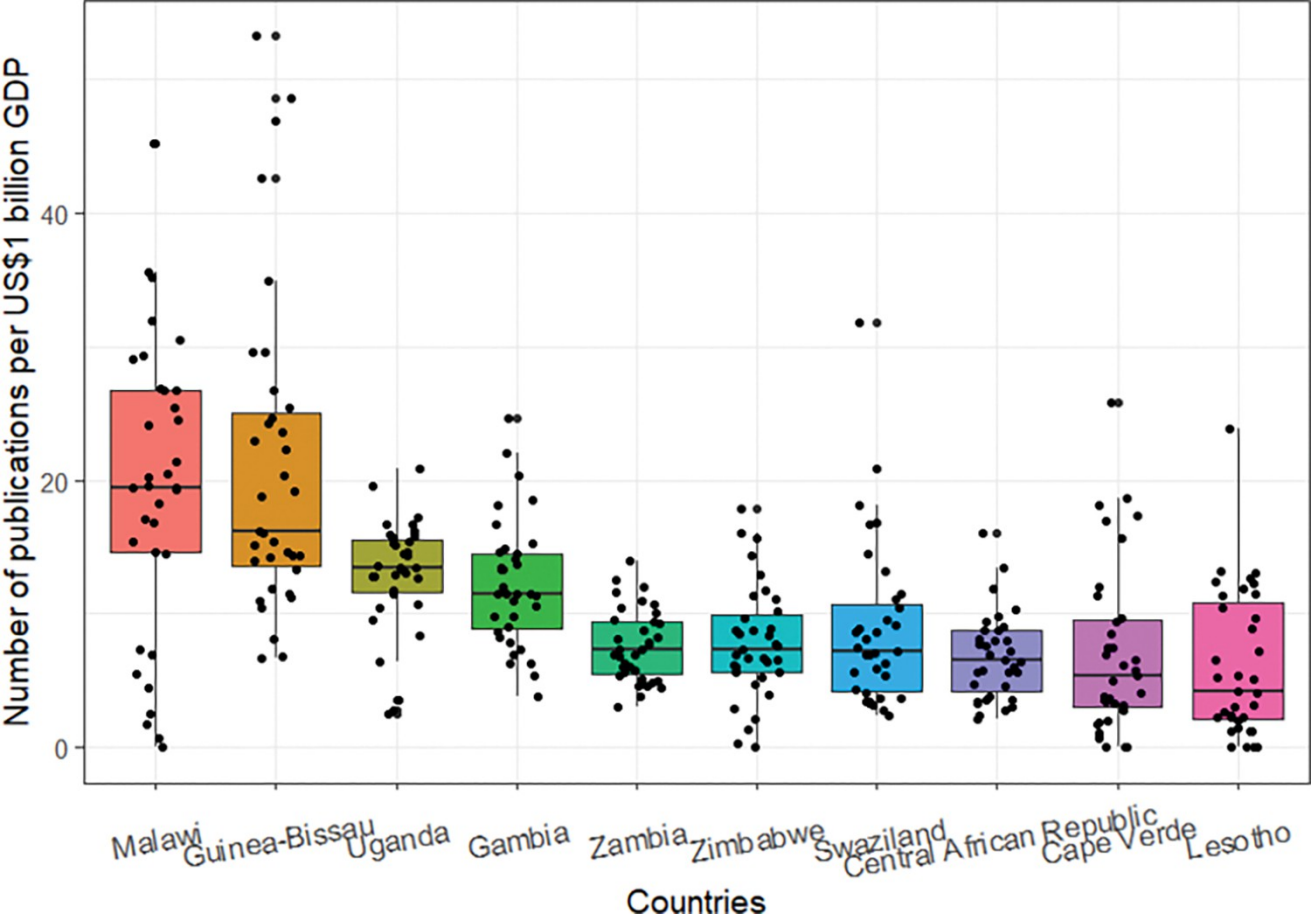
Top 10 most productive countries and the annual number of publications per US$1 billion GDP, 1986–2020.

The top 10 countries with total indexed publications relative to GDP are also ranked in Fig 10. Four countries, Malawi, Uganda, Zambia, and Zimbabwe, which are among the top 10 countries with the highest indexed publications, were also found to be among the most productive relative to GDP. The other six countries: Cape Verde, Central African Republic, Gambia, Guinea Bissau, Lesotho, and Swaziland, are relatively much less populous than the other four. All the countries belong to the low-income and lower-middle-income country groups.

## Discussion

### Main findings

Research findings must be published to contribute to the body of knowledge. This study’s objective was the description of HIV research output in Africa by country, beginning from when HIV was first announced as the official term for the virus that causes AIDS. PubMed remains the most widely used and optimal electronic database for biomedical research [25, 26].

Africa’s contribution to global health research output in 2014 was estimated at 1.3% [27]. Notably, in 2020, Africa accounted for 31% of global HIV research publications. Although this is markedly higher than Africa’s total contribution to global health research, this is relatively low for a continent with the highest burden of HIV infections. Africa’s proportion of HIV research output generally increased over the 35-year period, especially in the last 15 years. A study on the underrepresentation of authors from low and middle-income countries in research journals argued that authors from high-income countries with qualitatively and quantitatively significantly higher investments in research are at an advantage [28]. Another study found that the USA, U.K., and France were the leading countries with the most publications in the International Scientific Indexing (ISI) databases in 2003 [19, 29].

From our results, HIV research output differs across the various sub-regions in Africa. HIV research output was substantially higher in the Eastern, Southern, and Western African regions than in the Middle and Northern African regions. We found that the Republic of South Africa had the most research output, similar to findings in other analyses [22, 30]. Of note is that the four countries (Republic of South Africa, Uganda, Kenya, and Nigeria) that account for about 50% of total publications are anglophone. This disproportionate distribution in research output has been noted in multiple previous studies [22, 30]. The role of language in research output was outside the scope of the present study; however, it has been posited that non-English speaking authors are at a disadvantage with the dominance of English Language journals in the research world and the associated problem of language proficiency [26, 31–33]. The number and distribution of health research institutions and researchers in a country have also been highlighted as a crucial determinant of research output [27].

The factors associated with HIV research output in our analysis were economic strength (GDP per capita) and disease epidemiology (PLHIV burden). A positive correlation also exists between countries’ populations and research output. Associations between population and HIV research output have been previously reported [34]. In addition to the positive correlation between GDP and population, the higher research output from these populous African countries with relatively higher GDP (Republic of South Africa, Ethiopia, Nigeria, and Kenya) have been argued to be due to higher numbers of researchers and tertiary/research institutions [22, 30, 35–38].

Our study also showed a statistically significant correlation between the number of PLHIV in a country and HIV research output. These results align with the ‘burden of disease’ approach to research priorities and align with findings in studies by other researchers across different countries and specializations [39, 40]. A study of research intensity in infectious disease found that the large number of patients with HIV may have encouraged increased investment in research resources and motivated researchers to conduct studies about the disease [40]. Such increased investment in research priorities has led to recent advancements in evidence-based practice and policy formulation in HIV prevention, care, and treatment (e.g., Pre-exposure Prophylaxis and Prevention of Mother to Child Transmission-PMTCT) [41, 42].

Furthermore, while there was a consistent increase in the number of research publications across all regions over the years reviewed, there was a sharp spike in the number of publications between 2001–2005 across all regions, which served as the basis for the increasing surge of research publications. The establishment of The Global Fund to Fight AIDS, Tuberculosis and Malaria in 2002 and the President’s Emergency Plan for AIDS Relief (PEPFAR) in 2003- the largest funders of HIV programs in Africa could explain the spike in the number of research publications between 2001–2005 [43–45]. Although the U.S. and other development partners have been involved in addressing the global HIV crisis since the mid-1980s, the establishment of the Global Fund and PEPFAR in 2003 marked a pronounced increase in funding and attention to the HIV epidemic [43–45]. The association between research output and funding has been well documented by several studies [46–50].

The disparities in scholarly productivity and underrepresentation of Africa in global research output extend beyond the field of HIV. Similar findings have also been reported by studies that examined Africa’s COVID-19 research output [17, 27]. In particular, the studies buttressed the skewed research output favoring a few African countries and identified a trend in academic partnerships between countries with religious, language, or colonial ties [17, 27].

### Study limitations

A few limitations warrant comment. First, we limited the search to PubMed only, which may have prevented us from capturing relevant articles from other databases. Second, most articles indexed in PubMed are in English, which may have introduced bias in the selection of articles included in the analysis. For example, articles written in the official language of non-English speaking African countries would have been largely excluded from the PubMed search. Finally, due to limited resources and research infrastructure in many African countries, some publications are inaccessible through electronic databases. This could have also biased the selection of articles in our analysis. Despite these limitations, our search returned 83,527 articles included in the analysis.

## Conclusion

The study found that Africa’s contribution to global HIV research output increased over the 35-year period, but it remained relatively low compared to the continent’s burden of HIV infections. Factors associated with HIV research output, such as population size, economic strength, and disease epidemiology, can inform resource allocation to increase research productivity. Additionally, differences in HIV research output across various sub-regions in Africa and the disproportionate distribution of research output among a few countries highlight the need for more equitable partnerships. The marked increase in HIV research output between 2001 and 2005, associated with the establishment of the Global Fund and PEPFAR, emphasizes the importance of funding in promoting research productivity. Finally, our results on the underrepresentation of Africa in global HIV research output can inform policy decisions aimed at addressing these disparities in other fields, such as COVID-19 research.

## Supporting information

S1 Data (XLSX)

S1 File (R)
